# Nutraceutical Strategy to Counteract Eye Neurodegeneration and Oxidative Stress in *Drosophila melanogaster* Fed with High-Sugar Diet

**DOI:** 10.3390/antiox10081197

**Published:** 2021-07-27

**Authors:** Elisabetta Catalani, Giuseppina Fanelli, Federica Silvestri, Agnese Cherubini, Simona Del Quondam, Silvia Bongiorni, Anna Rita Taddei, Marcello Ceci, Clara De Palma, Cristiana Perrotta, Sara Rinalducci, Giorgio Prantera, Davide Cervia

**Affiliations:** 1 Department for Innovation in Biological, Agro-Food and Forest Systems (DIBAF), Università degli Studi della Tuscia, largo dell’Università snc, 01100 Viterbo, Italy; ecatalani@unitus.it (E.C.); federica.silvestri@unitus.it (F.S.); agnese.cherubini@studenti.unitus.it (A.C.); simona.delquondamgiuseppedettocelli@studenti.unitus.it (S.D.Q.); 2 Department of Ecological and Biological Sciences (DEB), Università degli Studi della Tuscia, largo dell’Università snc, 01100 Viterbo, Italy; giuseppina.fanelli@unitus.it (G.F.); bongiorni@unitus.it (S.B.); m.ceci@unitus.it (M.C.); sara.r@unitus.it (S.R.); prantera@unitus.it (G.P.); 3 Department of Agriculture and Forest Sciences (DAFNE), Università degli Studi della Tuscia, via San Camillo de Lellis snc, 01100 Viterbo, Italy; 4 Section of Electron Microscopy, Great Equipment Center, Università degli Studi della Tuscia, largo dell’Università snc, 01100 Viterbo, Italy; artaddei@unitus.it; 5 Department of Medical Biotechnology and Translational Medicine (BioMeTra), Università degli Studi di Milano, via L. Vanvitelli 32, 20129 Milano, Italy; clara.depalma@unimi.it; 6 Department of Biomedical and Clinical Sciences “Luigi Sacco” (DIBIC), Università degli Studi di Milano, via G.B. Grassi 74, 20157 Milano, Italy; cristiana.perrotta@unimi.it

**Keywords:** nutraceuticals, *Drosophila melanogaster*, neurodegeneration, diabetic retinopathy, hyperglycemic damage, retina, visual functions, oxidative stress, apoptosis, autophagy

## Abstract

Aberrant production of reactive oxygen species (ROS) is a common feature of damaged retinal neurons in diabetic retinopathy, and antioxidants may exert both preventive and therapeutic action. To evaluate the beneficial and antioxidant properties of food supplementation with Lisosan G, a powder of bran and germ of grain (*Triticum aestivum*) obtained by fermentation with selected lactobacillus and natural yeast strains, we used an in vivo model of hyperglycemia-induced retinal damage, the fruit fly *Drosophila melanogaster* fed with high-sucrose diet. Lisosan G positively affected the visual system of hyperglycemic flies at structural/functional level, decreased apoptosis, and reactivated protective autophagy at the retina internal network. Also, in high sucrose-fed Drosophila, Lisosan G reduced the levels of brain ROS and retina peroxynitrite. The analysis of oxidative stress-related metabolites suggested 7,8-dihydrofolate, uric acid, dihydroorotate, γ-L-glutamyl-L-cysteine, allantoin, cysteinyl-glycine, and quinolate as key mediators of Lisosan G-induced inhibition of neuronal ROS, along with the upregulation of glutathione system. Of note, Lisosan G may impact oxidative stress and the ensuing retinal cell death, also independently from autophagy, although the autophagy-ROS cross-talk is critical. This study demonstrated that the continuous supplementation with the alimentary integrator Lisosan G exerts a robust and multifaceted antioxidant effect on retinal neurons, thus providing efficacious neuroprotection of hyperglycemic eye.

## 1. Introduction

Retinal cells are extremely metabolically active and enriched of mitochondria, the predominant intrinsic sources of reactive oxygen species (ROS) [1,2]. In addition, as a result of its anatomy and physical location, the retina is exposed to high levels of light and other electromagnetic radiations, which are potent generators of ROS [1,3]. ROS are produced at moderate levels during normal metabolism, as products of cellular metabolism required in maintaining retinal functions. In contrast, excessive ROS production resulting from mitochondrial dysfunction and altered antioxidant defense contribute to several pathophysiological events. In other words, the oxidative imbalance between formation and clearance of ROS impairs survival signaling, thus playing a key role in retinal disease progression, such as diabetic retinopathy (DR) [4,5,6,7].

In eukaryotic cells, antioxidative defense mechanisms maintain the physiological balance between ROS generation and clearance. However, a state of oxidative stress occurs when ROS overproduction overwhelms the intrinsic antioxidant capacity of cells, resulting in damage to biomolecules. Antioxidants act at various levels: preventing ROS formation, scavenging free radicals, or enhancing antioxidant defenses. Agents with antioxidant mechanisms of action may exert both preventive and therapeutic action in retinopathies and DR [4,5,6], the major cause of blindness for patients of working age. In this respect, there is compelling evidence that hyperglycemia-induced production of ROS promotes microvascular complications, neurodegeneration, and angiogenesis, all of which contribute to the pathology that occurs in DR [5,6,7]. Of notice, in DR, the damage of retinal neurons may be appreciated before clinical symptoms, thus suggesting that early abnormalities in the neuroretina, triggered by hyperglycemia, could promote vascular impairments as a subsequent event [7,8,9,10,11]. Although the mechanisms by which ROS influence neuronal damage in DR are still not fully understood [4,5,6], the most important histological feature during hyperglycemia and DR at early stages is neuronal apoptosis [7,8,9]. Alterations of the catabolic pathway autophagy also play a key role in different retina dysfunctions, including DR [5,11,12,13,14,15,16,17,18,19,20,21]. Recently, we used the fruit fly *Drosophila melanogaster* fed with high-sucrose diets as an in vivo model of hyperglycemia-induced neuronal damage [22]. This model facilitates the exploration and treatments of the early degenerative features affecting the retina during DR at both functional (visual performances) and molecular/cellular level (eye neurodegeneration, neuronal apoptosis/autophagy dysregulation, and oxidative stress), thus complementing more traditional vertebrate tools [23].

Clinical medications or surgeries approved to treat DR are used for the late stages of disease progression [24]. However, there is a clear need for new strategies acting early at the molecular or cellular level to prevent DR development. Indeed, a poor visual prognosis is offered for the early stages of DR since no management is given other than glycemic control. In this respect, therapeutic options based on the management of oxidative stress and its progression could be part of the timely treatment of DR [5,6,7]. From a general point of view, nutraceuticals act on multiple intracellular signals and are considered as positive neuroprotectants of retinal cells. In particular, both in vitro and in vivo evidence demonstrated the significative antioxidant properties of a wide variety of individual foods, macro or micronutrients, dietary supplements, and dietary patterns, which may inhibit the early diabetes-driven molecular mechanisms responsible for the onset and progression of DR [25,26,27].

The nutritional supplement Lisosan G is a fermented powder obtained from organic whole grains (*Triticum aestivum*) enriched in bioactive substances [28]. The protecting effects of Lisosan G on different models have been associated with radical scavenging, a decrease of oxidative stress, and activation of antioxidant factors [28,29,30,31,32,33]. In the nervous system, Lisosan G administered by oral gavage exhibited protective neurovascular effects on diabetic retinas of rats treated with streptozotocin and in mouse retina ex-vivo explants challenged with oxidative stress [34]. These results prompted us to use a nutraceutical approach in *D. melanogaster* to evaluate in detail the antioxidant properties of Lisosan G and its beneficial actions on glucose-induced retinal neurodegeneration. Flies were fed with high-sucrose diets and observed after diet supplementation with Lisosan G. The visual system was then characterized at structural, functional, and molecular/cellular levels as an in vivo model mimicking the neuronal defects occurring in the human diabetic eye at an early stage.

## 2. Materials and Methods

### 2.1. Reagents

The alimentary integrator Lisosan G, which is registered with the Italian Ministry of Health as a nutritional supplement, was obtained from Agrisan Company (Larciano, Pistoia, Italy). Lisosan G is a powder obtained by fermentation of bran and germ of grain (*Triticum aestivum*). As previously detailed [30], the manufacturing process consists of grounding the wholegrain to a rough powder. The bran and germ are then collected separately from this preliminary product before water supplementation to moisten the mixture. Fermentation begins through inoculation of selected lactobacillus and natural yeast strains as microbic starting cultures. Once the product is sufficiently fermented, it is dried to obtain Lisosan G powder. Lisosan G contains at least 20 major components, including proteins, lipids, glucids, polysaccharides, oligoelements (e.g., phosphate, sulphur, magnesium, iron, zinc, copper, and selenium), vitamins (e.g., vitamin E, B1, B2, B6, and nicotinamide), and fatty acid (e.g., linoleic acid, linolenic acid, and oleic acid) [28]. Lisosan G appears particularly rich in antioxidant components such as phenolic components (flavonoids and flavonols) and alpha-lipoic acid [28,29,30,31,32].

Bovine serum albumin (BSA), normal goat serum and Alexa Fluor secondary antibodies were purchased from Invitrogen-ThermoFisher Scientific (Monza, Italy). Fluorescent phalloidin (iFluor 555, #ab176756) was obtained from Abcam (Cambridge, UK). Agar 100 resin/propylene oxide and Agar 100 resin were obtained from Electron Microscopy Sciences (Hatfield, PA, USA). Primary antibodies: anti-cleaved caspase 3 (#9664) was purchased from Cell Signaling Technology (Danvers, MA, USA); anti-nitrotyrosine (#A-21285) was obtained from Invitrogen-ThermoFisher Scientific (Monza, Italy); anti-Light-Chain 3 (LC3) (#ab128025) and anti-p62/Sequestosome-1 (SQSTM1) (#P0067) were purchased from Sigma-Aldrich (St. Louis, MO, USA). All other chemicals, including Liquid Chromatography–Mass Spectrometry (LC-MS) grade acetonitrile, chloroform, methanol, formic acid, and water (≥98% chemical purity), were from Sigma-Aldrich (St. Louis, MO, USA).

### 2.2. Fly Husbandry

All experiments were performed with female and male adult *D. melanogaster* (Oregon-R strain from Bloomington Drosophila Stock Center, Indiana University Bloomington, IN, USA). As previously reported [20,22,35], flies were routinely raised on a standard cornmeal agar food (pH 5.5) at 25 °C with minor modifications. Fly food was prepared as follows: 100 g of yellow cornmeal, 100 g of brewer’s yeast, 8 g of agar, and 75 g of sucrose (5% *w*/*v*) were mixed and dissolved by adding warm plain water to a final volume of 1.5 l, the hydration source of the flies. The mixture was autoclaved and allowed to cool down slowly. The broad-spectrum fungicide Nipagin (3 g dissolved in 16 mL of absolute ethanol) was added when the temperature reached approximately 50 °C, and the mixture was then dispensed into vials. Populations of adult flies (3 days old) were placed in vials (15 females and 10 males) for mating and egg laying. After 3 days, mating flies were removed and, at around day 10 from mating, adults emerged from their pupal cases (eclosion).

### 2.3. Treatments

Adult Drosophila (5–6 days old) were transferred for 10 days on either the standard food (5% sucrose, corresponding to 0.146 M) or the high-sucrose diet (35% *w*/*v*, 1.022 M), obtained changing sucrose independently while keeping the other components constant. As previously detailed [22], standard food of flies contained carbohydrates and protein/fat reservoir; supplemental sucrose increased carbohydrate energy at 90% of calories from carbohydrates (standard food: 72%). Where indicated, flies were reared on diets supplemented with Lisosan G. A preliminary dose–response of Lisosan G was tested in hyperglycemic Drosophila using 300, 100, and 10 µg/mL [34] for 10 days, evaluating general behaviors of flies and their response to light. The highest concentration appeared somewhat toxic at visual observations, while 100 and 10 μg/mL of Lisosan G resulted in the comparable recovery of phototaxis performance. Therefore, subsequent experiments were performed using Lisosan G at a final concentration of 10 µg/mL. Where indicated, flies were also reared on a high-sucrose diet supplemented with 10 nM rapamycin as previously reported [20], both in the absence and in the presence of Lisosan G.

### 2.4. Fluorescence Microscopy

Using published protocols [20,22,35,36], *D. melanogaster* heads were immersion-fixed overnight or for 48 h, in 4% paraformaldehyde in 0.1 M Phosphate Buffer (PB) at 4 °C, transferred to 12% sucrose in PB and stored at 4 °C for at least 24 h. Longitudinal and cross sections (10 µm) were obtained by a cryostat, mounted onto positively charged slides and stored at −20 °C until use. To allow proper comparison in the retina/eye, the same depth/region of the structure was sectioned. Fluorescent phalloidin (F-actin staining, 1:2500) was used for observation with: (i) Axioskop 2 plus conventional microscope (Carl Zeiss, Oberkochen, Germany) equipped with the Axiocam MRC photo camera and the Axiovision software (longitudinal sections); and (ii) LSM 710 confocal microscope (Carl Zeiss, Oberkochen, Germany) (cross sections).

For immunostaining detection, longitudinal sections were washed in PB and then pre-incubated for 30 min at room temperature with 5% BSA and 10% of normal goat serum in PB containing 0.5% Triton X-100. Pre-treated sections were incubated overnight at 4 °C with one of the following rabbit primary antibodies: anti-nitrotyrosine (1:100), anti-cleaved caspase 3 (1:500), anti-LC3 (1:100), and anti-p62/SQSTM1 (1:200) [20,37,38,39,40,41,42,43,44] in PB containing 0.5% Triton X-100. Following washes in PB, the sections were incubated in the appropriate Alexa Fluor secondary antibodies (1:200) in PB for 1.5 h at room temperature. Incubation in secondary antibody alone was performed as a negative control. Images were acquired by a Zeiss LSM 710 confocal microscope. The analysis of nitrotyrosine, cleaved caspase 3, LC3, and p62 immunostaining was carried out on the single images of each eye section. Each image was converted to grayscale and normalized to the background using Adobe Photoshop (Adobe Systems, Mountain View, CA, USA). Mean gray levels were then measured in the selected areas [45].

### 2.5. Transmission Electron Microscopy (TEM)

*D. melanogaster* heads samples were fixed and dehydrated in agreement with previous studies [20,22,46]. The dehydration was followed by two steps in pure propylene oxide for 10 min each, at 4 °C. Samples were then infiltrated with mixtures of Agar 100 resin/propylene oxide in different percentages. At the end of the procedure, samples were embedded in pure Agar 100 resin and let to polymerize for 2 days at 60 °C. Resin blocks were cut with Reichert Ultracut ultramicrotome using a diamond knife. Ultrathin sections (60–80 nm) (Leica Microsystems, Wetzlar, Germany) were collected on copper grids, stained with uranyl acetate and lead citrate, and observed with a JEOL 1200 EXII electron microscope (Jeol, Tokyo, Japan). Micrographs were captured by the Olympus SIS VELETA CCD camera equipped with iTEM software (Olympus, Tokyo, Japan). Quantitative analysis of photoreceptor cell size was performed on selected TEM images of Drosophila retinas. In particular, two representative images at the same magnification were examined from each eye section. The cell area of photoreceptors from at least 5 different ommatidia/eyes was measured by Image J software.

### 2.6. Body Weight and Glucose Analysis

Flies were collected in screw cap tubes and weighed in groups of 10 using an ultramicro balance (with a high resolution of up to 0.0001 mg). For glucose measurements, samples were analyzed in agreement with previous reports [22]. Briefly, metabolite extraction was performed on fly samples by water/methanol/chloroform mixture (Bligh-Dyer method). Following mixing and centrifugation, fractions were transferred to Eppendorf tubes and then dried at 4 °C before resuspension in ultrapure water and analysis by LC-MS. Chromatographic separation was carried out on a Reprosil C18 column (2.0 mm × 150 mm, 2.5 μm—Dr. Maisch, Ammerbuch-Entringen, Germany) with a 0–100% linear gradient of solvent A (double-distilled 18 mΩ water, 10 mm ammonium acetate) to B (100% acetonitrile, 10 mm ammonium acetate) at a flow rate of 0.2 mL/min. Column flow was directed into the mass spectrometer (Q Exactive ThermoFisher Scientific, Monza Italy) operating in negative ion mode and scanning in full MS mode (2 μscans) at 70,000 resolution from 60 to 1000 *m*/*z*. Glucose concentrations were calculated via a six-point standard curve and related to the corresponding flyweights.

### 2.7. Determination of ROS

As previously published with minor modifications [47], one hundred head’s fly per experimental group were weighted and then homogenized in 1000 μL of 10 mM Tris-buffer, pH 7. The homogenates were centrifuged at 1.000× *g* for 5 min at 4 °C, and 100 μL of each supernatant were incubated in a 96 multiwell plate in the presence of 5 µM 2′,7′-Dichlorofluorescin diacetate (DCFH-DA) at 37 °C for 60 min. Two-electron oxidation of DCFH results in the formation of the fluorescent product DCF, which was recorded at the end of the incubation at an excitation wavelength of 488 nm and an emission wavelength of 525 nm in a DTX 880 Multimode Detector (Beckman Coulter, Brea, CA, USA).

### 2.8. Metabolite Analysis

Metabolites were extracted from at least one hundred head’s fly per experimental group. As previously published [22], samples were lysed in 0.2 mL of ice-cold ultra-pure water (18 MQ). The tubes were plunged into dry ice or a circulating bath at −25 °C for 0.5 min and then into a water bath at 37 °C for 0.5 min. To each tube, 0.6 mL of −20 °C methanol and then 0.4 mL of −20 °C chloroform were added. The tubes were mixed every 5 min for 30 min. Subsequently, each tube was centrifuged at 1000× *g* for 1 min at 4 °C, before being transferred to −20 °C for 2–8 h. The solutions were then centrifuged for 15 min at 15,000× *g* for 10 min at 4 °C, and the collected supernatants were dried. Finally, the dried samples were re-suspended in 0.1 mL of water and transferred to glass autosampler vials for LC-MS analysis. Supernatants were injected into an ultra-high-pressure liquid chromatography system (Ultimate 3000) coupled online with a Q Exactive mass spectrometer (ThermoFisher Scientific, Monza, Italy) operating in positive ion mode and scanning in full MS mode (2 μscans) at 70,000 resolution from 60 to 1000 *m*/*z*. Chromatographic separations were achieved with a Reprosil C18 column (2.0 mm × 150 mm, 2.5 μm—Dr. Maisch, Ammerbuch-Entringen, Germany) working at a temperature of 30  °C and a flow rate of 0.2 mL/min. For metabolite elution, a 0–100% linear gradient of solvent A (ddH_2_O, 0.1% formic acid) to B (acetonitrile, 0.1% formic acid) was employed over 20 min returning to 100% A in 3 min. Data files of replicates were processed by MAVEN. 8.1 (http://maven.princeton.edu/index.php) upon conversion of raw files into mzXML format through MassMatrix (Cleveland, OH, USA).

### 2.9. Phototaxis Assay

Response to light was assessed as reported before [20,22]. Briefly, a plastic vial (2.5 cm × 9.5 cm) with *D. melanogaster* was inserted and connected to a glass tube (3.0 cm × 23.0 cm) by transparent tape. The transparent apparatus (30 cm) was placed horizontal and perpendicular to the light source. The directional light source from one side, placed horizontally 15 cm away from the tube, acted as an attractant for the flies. In a dark room, 10–30 flies were independently introduced in the apparatus and left separately for 30 min. This allowed the adaptation of the flies to darkness. The apparatus was then gently pounded down to place the flies at the opposite end from the light. The light was then turned on, and a timer was started. A camera was recording fly behavior and their movement (horizontal walking) towards the light source during the experiment (2 min). Each trial was performed three times, at 1 min intervals, and the results were averaged. For the analysis of visual responses, the flies were counted at 10, 20, 30, 40, 50, 60, and 120 s for each marked part of the apparatus, i.e., 0–10 cm (the chamber nearest to origin), 11–20 cm (the chamber next furthest to origin), and 21–30 cm (the chamber furthest to origin).

### 2.10. Statistics

Statistical significance of raw data between the groups in each experiment was evaluated using unpaired Student’s *t*-test (single comparisons) or one-way ANOVA followed by the Tukey post-test (multiple comparisons). A *p* value ≤ 0.05 was considered statistically significant. The GraphPad Prism software package (GraphPad Software, San Diego, CA, USA) was used. Metabolomics data were analyzed by the MetaboAnalyst online tool (https://www.metaboanalyst.ca/, accessed on 15 June 2021). Multivariate partial least squares discriminant (PLS-DA) and variable of importance in prediction (VIP) analysis were performed. Also, univariate one-way ANOVA followed by Fisher’s least significant difference (LSD) test was carried out, and false discovery rate (FDR) was used for controlling multiple testing (*p*-value FDR cut off 0.05). Data belonging to different experiments were represented and averaged in the same graph. The results were expressed as means ± SEM of the indicated *n* values.

## 3. Results

### 3.1. Eye Organization

**Figure 1:**
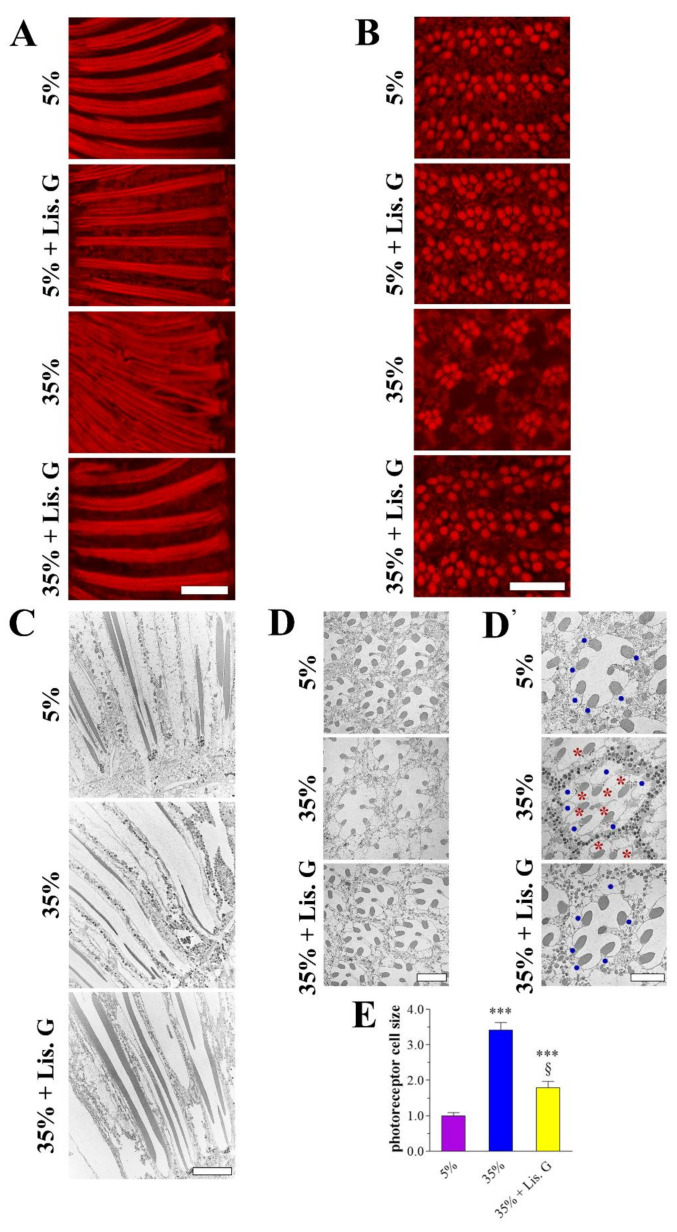
Eye phenotype of adult *D. melanogaster* after 10 days feeding with 5% sucrose diet (control, normoglycemic flies) or supplemented with 35% sucrose (hyperglycemic flies), in the absence and in the presence of Lisosan G at 10 µg/mL. (**A**) Conventional (longitudinal sections) and (**B**) confocal (cross sections) microscopy analysis of Drosophila eyes stained with fluorescent phalloidin (F-actin staining) to detect rhabdomere morphology and the pattern of ommatidia and rhabdomeres, respectively. Scale bars: 20 μm (longitudinal) and 10 μm (cross). Images are representative of at least *n* = 30 animals obtained from 5 independent experiments. (**C**) Retina ultrastructure of columnar pattern by TEM analysis (longitudinal sections). Scale bar: 10 μm. (**D**) TEM micrographs of ommatidial cross sections. Scale bar: 5 μm. (**D’**) The higher magnification of different fields highlighted the alteration (size) of photoreceptor cell bodies (blue dots) and the presence of vacuoles (red asterisks). Please note that in the electron microscopic preparations, fixation and sectioning often led to the loss of the black pigment granule content that therefore appeared only in some images. Scale bar: 5 μm. (**E**) Quantitative analysis of photoreceptor cell size. Results are expressed by setting the mean cell area of 5% normoglycemic control as 1. *** *p* < 0.0001 vs. 5% control; § *p* < 0.01 vs. 35% sucrose. Images and data are representative of at least *n* = 15 animals obtained from 4 independent experiments.

The increased availability of dietary sucrose for 10 days in adult *D. melanogaster* induced hyperglycemia and eye neurodegeneration, without significant effects on growth and viability [22]. To evaluate whether Lisosan G positively affected the visual system of hyperglycemic animals, adult Drosophila were raised for 10 days with 35% sucrose regimens both in the absence and in the presence of Lisosan G at a concentration of 10 µg/mL, similar to previous reports in ex vivo mouse retinas [34]. The array of ommatidia and rhabdomeres, the actin-rich apical portion of the photoreceptor containing microvilli and light-sensing proteins (labeled with phalloidin) were observed by fluorescence microscopy in longitudinal and cross sections of the Drosophila eye. Lisosan G did not alter the regular pattern and the rhabdomere morphology of the control normoglycemic (standard diet—5% sucrose) eye, while it rescued the phenotype of flies raised with 35% sucrose. Specifically, the abnormal aspect of rhabdomere columns (Figure 1A), the disorganization of the hexagonal ommatidia profile, and the altered spaces between adjacent ommatidia (Figure 1B), such that their rhabdomeres appeared closer, which are all hallmarks of hyperglycemic fly eye damage [22], were not detectable after Lisosan G administration. TEM microscopy in ultrathin longitudinal and cross sections confirmed this scenario, also showing the presence of numerous vacuole-like structures in 35% sucrose group but not in Lisosan G-treated samples (Figure 1C,D). In addition, the increase of photoreceptor size achieved in hyperglycemic eyes was partially restored after treatment of flies with Lisosan G (Figure 1D,E).

### 3.2. Body Weight and Glycemia

To evaluate whether Lisosan G affected systemic glucose in adult Drosophila, at 10 days of treatment with 5% control or 35% sucrose regimens, flies were weighed, harvested, and the whole-body glucose quantified. As previously published [22], no significant differences in body weight were observed between standard and high-sucrose groups (Figure S1A), while 35%-treated animals were hyperglycemic (Figure S1B). Of notice, Lisosan G (10 µg/mL) did not affect the body weight and glucose content of flies.

### 3.3. Oxidative Stress: Peroxynitrite and ROS

**Figure 2:**
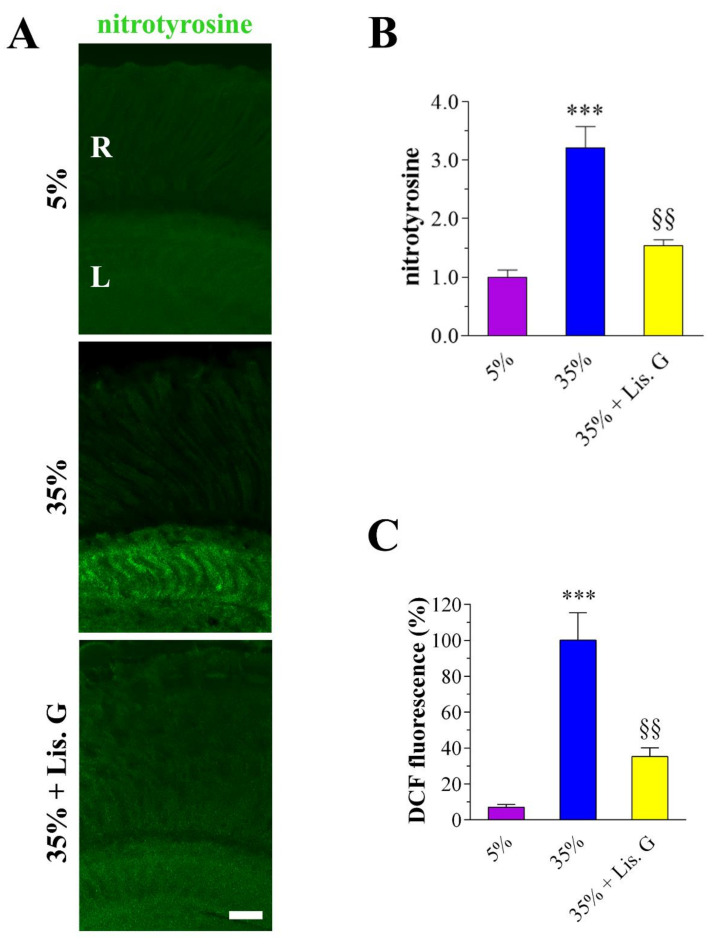
Oxidative stress in adult *D. melanogaster* after 10 days feeding with 5% sucrose diet (control, normoglycemic flies) or supplemented with 35% sucrose (hyperglycemic flies), in the absence and in the presence of Lisosan G at 10 µg/mL. (**A**) Confocal microscopy immunofluorescence imaging of nitrotyrosine in retina sections. Scale bar: 20 μm. R: retina, L: lamina. (**B**) Quantitative analysis of nitrotyrosine immunofluorescence. Results are expressed as arbitrary units. Images and data are representative of at least *n* = 30 animals obtained from 5 independent experiments. (**C**) Measurements of ROS in Drosophila heads by DCF fluorescence intensity. Results are expressed as a percentage of 35% sucrose. Data are representative of at least *n* = 300 animals obtained from 3 independent experiments. *** *p* < 0.0001 vs. 5% control; §§ *p* < 0.001 vs. 35% sucrose.

The antioxidants effects of Lisosan G on hyperglycemic damaged retinas were verified first by confocal immunostaining using an anti-nitrotyrosine antibody to detect peroxynitrite. Elevated nitrotyrosine immunostaining was clearly detected in the lamina of adult Drosophila eye under high-sucrose regimens (Figure 2A) [22]. Noteworthy, in 35%-treated flies exposed to Lisosan G (10 µg/mL, 10 days), peroxynitrite labeling significantly decreased to values comparable with 5% normoglycemic control (Figure 2B).

Oxidative stress was then determined using the DCFH-DA probe. In fly brains of Drosophila, the levels of DCF fluorescence induced by high-sucrose regimens were restored by Lisosan G, indicating a significant reduction of ROS generation (Figure 2C).

### 3.4. Redox Metabolites

**Figure 3:**
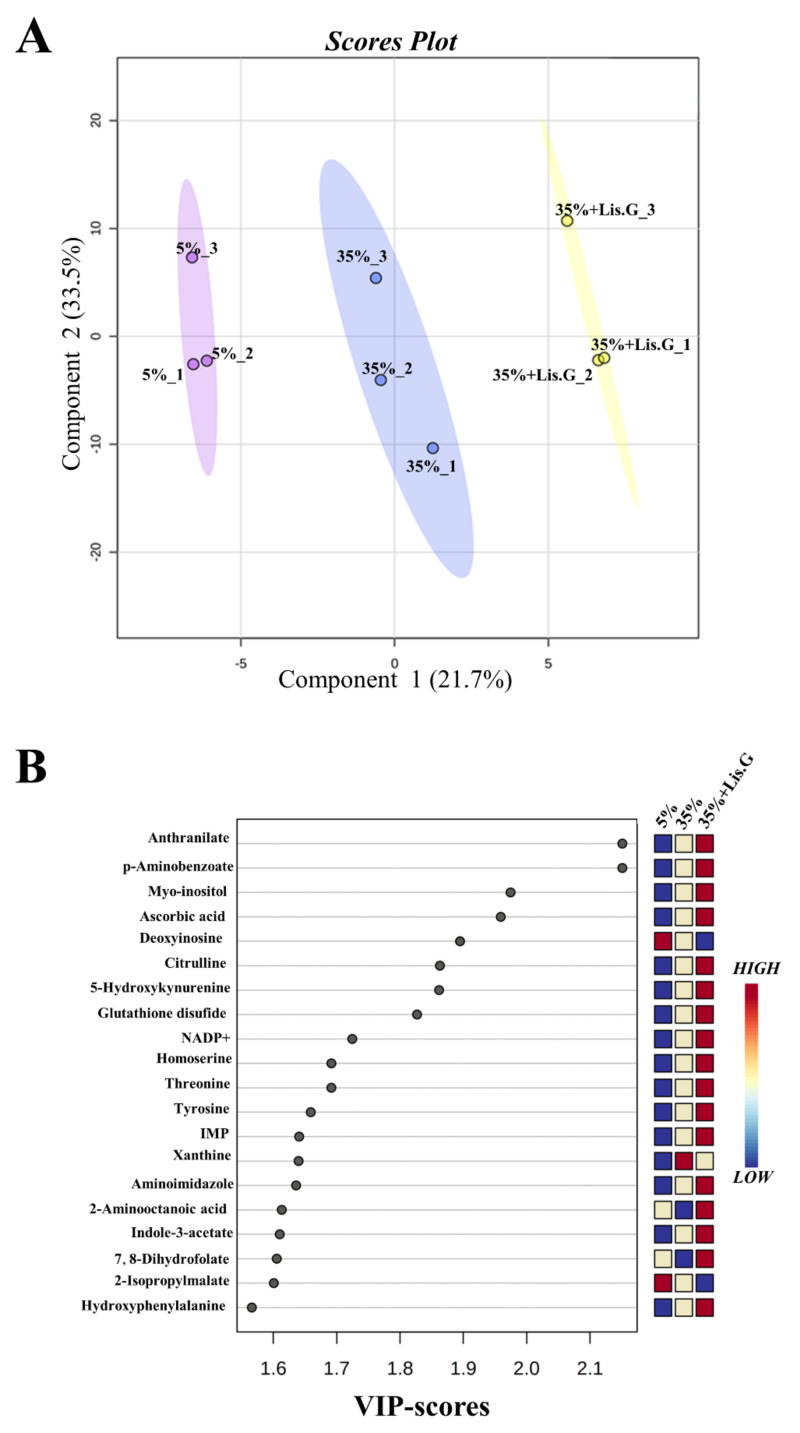
Oxidative stress-related metabolites in adult heads of *D. melanogaster* after 10 days feeding with 5% sucrose diet (control, normoglycemic flies) or supplemented with 35% sucrose (hyperglycemic flies), in the absence and in the presence of Lisosan G at 10 µg/mL. (**A**) PLS-DA score plot. Triplicate samples within each group, i.e., 1 = 5% sucrose (purple), 2 = 35% sucrose (blue), and 3 = 35% sucrose + Lisosan G (yellow) are indicated by numbers. X-axis and Y-axis are labeled with the first principal component and the second principal component explaining 21.7% and 33.5% of the total variation, respectively. (**B**) VIP score plot. Colored boxes indicate the relative concentrations of the corresponding metabolite in each group under the current study (red, up-regulation; blue, down-regulation). Data are representative of 3 replicate samples from at least *n* = 100 animals.

**Figure 4:**
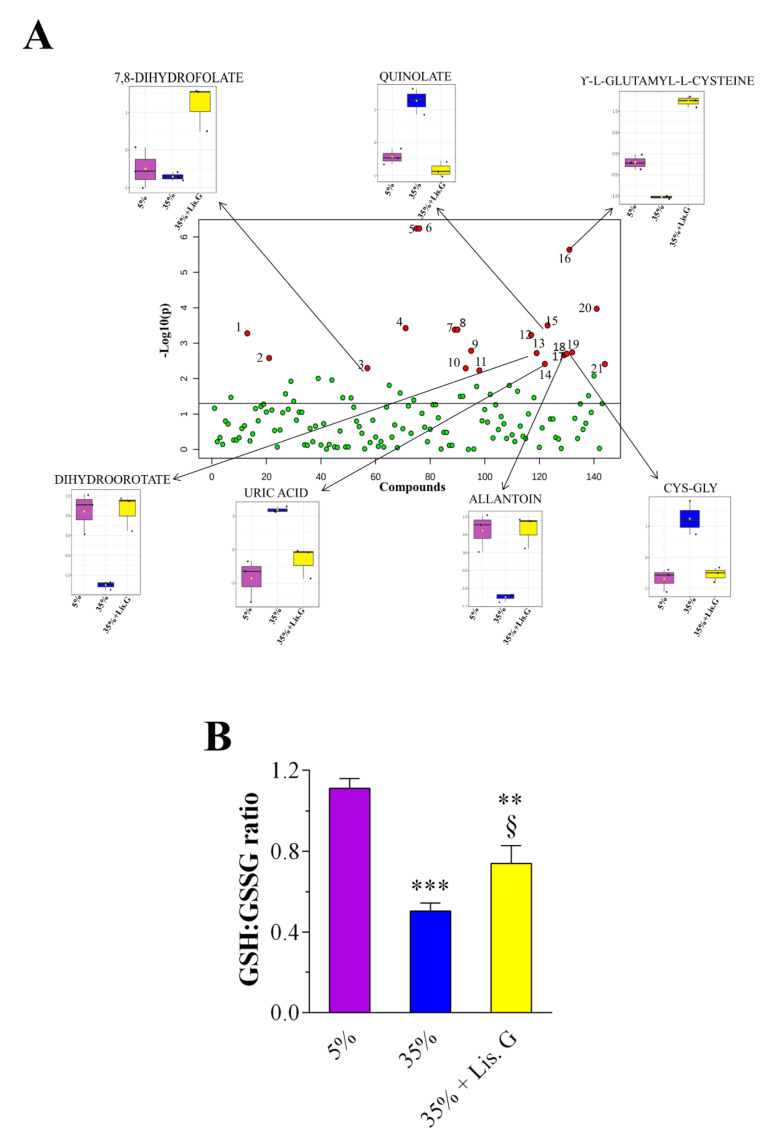
Changes of metabolome in adult heads of *D. melanogaster* after 10 days feeding with 5% sucrose diet (control, normoglycemic flies) or supplemented with 35% sucrose (hyperglycemic flies), in the absence and in the presence of Lisosan G at 10 µg/mL. (**A**) one-way ANOVA scatter plot. X-axis depicts the total number of metabolites detected. Red circles illustrate metabolites that showed statistically significant differences (FDR adjusted *p* < 0.05). Box plots depict the levels of most impacted metabolites related to oxidative stress in the experimental groups. (**B**) GSH:GSSG ratio. *** *p* < 0.0001 and ** *p* < 0.001 vs. 5% control; § *p* < 0.01 vs. 35% sucrose. Data are representative of 3 replicate samples from at least *n* = 100 animals.

These data prompted us to further dissect the oxidative stress-related machinery modulated by Lisosan G in our system. We performed an untargeted metabolomics analysis to identify changes in metabolites linked to cellular redox state. A total of 146 metabolites was annotated. Multivariate PLS-DA statistical analysis was carried out (Figure 3A). Results revealed a distinct separation among the sample groups (5%, 35%, and 35% sucrose + Lisosan G-treated flies), indicating that their metabolomic profiles are clearly distinguishable. R2 and Q2 values were thus calculated as measures of prediction accuracy. Satisfactory modeling and prediction results were already gained with three principal components (accuracy = 1, R2 = 0.99, Q2 = 0.87; data not shown). The VIP score was then utilized to select the most discriminative metabolites, as reported in Figure 3B. A metabolite with a VIP score greater than or equal to 1.0 was considered a variable that highly contributed to the observed separation within the PLS-DA plot. To determine the significantly up- and down-regulated metabolites in trios, a one-way ANOVA statistical test was conducted by Fisher’s LSD method (FDR adjusted *p* < 0.05; Table 1), which finally identified 21 most significant features between the samples. Among them, we detected seven statistically differentially abundant metabolites, i.e., 7,8-dihydrofolate (7,8-DHF), uric acid, dihydroorotate (DHO), γ-L-glutamyl-L-cysteine (γ-GC), allantoin, cysteinyl-glycine (Cys-Gly), and quinolate, which are directly or indirectly linked to ROS (Figure 4A). In detail, 7,8-DHF, γ-GC, DHO, and allantoin decreased in flies raised under a 35% sucrose regimen, whereas quinolate, uric acid, and Cys-Gly strongly increased. Of interest, in the presence of Lisosan G uric acid, DHO, allantoin, Cys-Gly, and quinolate reached values comparable to 5% normoglycemic control, while the levels of 7,8-DHF and γ-GC were found even higher.

Finally, we calculated the intracellular concentrations of the reduced form of glutathione (GSH) and oxidized form of GSH (GSSG) (Figure S2) since the GSH:GSSG ratio is considered a sensitive marker of oxidative stress and its chronic decrease reflects a reduced antioxidant capacity/increased vulnerability to oxidative damage. As shown in Figure 4B, GSH:GSSG ratio was significantly lower in 35% sucrose-treated flies when compared to the 5% group. Of notice, the administration of Lisosan G to hyperglycemic flies increased the ratio of GSH and GSSG.

### 3.5. Vision Behavior

**Figure 5:**
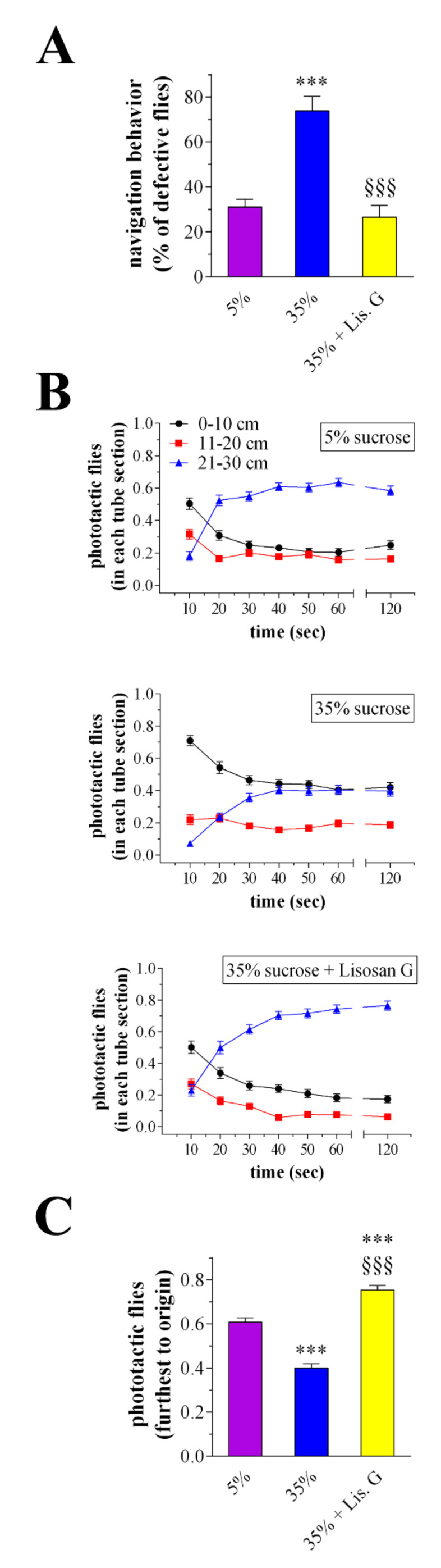
Phototaxis assay of adult *D. melanogaster* after 10 days feeding with 5% sucrose diet (control, normoglycemic flies) or supplemented with 35% sucrose (hyperglycemic flies), in the absence and in the presence of Lisosan G at 10 µg/mL. (**A**) Analysis of Drosophila navigation strategies. Results are expressed as the percentage of flies exhibiting a defective behavior within each experimental group. (**B**) Drosophila were counted at 10, 20, 30, 40, 50, 60, and 120 s for each marked part of the apparatus, i.e., 0–10 cm (the chamber nearest to origin), 11–20 cm (the chamber next furthest to origin), and 21–30 cm (the chamber furthest to origin). (**C**) Drosophila in the chamber furthest to origin were counted at 60–120 s. Results are expressed as the percentage of total flies in the chambers at each time points. *** *p* < 0.0001 vs. 5% control; §§§ *p* < 0.0001 vs. 35% sucrose. Data are representative of at least *n* = 200 animals obtained from 10 independent experiments run in triplicate.

When given a choice, adult D. *melanogaster* were attracted by light, avoiding shaded areas. The investigation of visual system response by a phototaxis assay may thus provide a functional correlation to a structural/molecular alteration of an eye [20,22,23]. In this respect, the navigation behavior of flies was first evaluated as an index of vision response. A normal behavior consists of flies that move straight towards the light source, while a defective behavior consists of motionless animals and those moving perpendicular to the light or unbiased towards and away from the light. Accordingly to previous observations [22], the number of impaired Drosophila increased at 10 days of high-sucrose feeding. Consistent with the amelioration of the eye homeostasis, treatment with 10 µg/mL Lisosan G re-established the defective:normal proportion of flies to values comparable with 5% normoglycemic control (Figure 5A). During the assay, the improvement of Drosophila’s responsiveness to the light by Lisosan G was also achieved when flies were counted at different time points for each chamber, i.e., nearest, next furthest, and furthest from the origin (Figure 5B). In particular, many flies raised under 35% sucrose regimen remained in the chamber next to the origin, and those reaching the light did it more slowly than 5%-treated animals [22]. Of interest, the administration of Lisosan G ameliorated the light response of hyperglycemic flies robustly. As shown in Figure 5C, 35% sucrose + Lisosan G-treated Drosophila counted in the chamber furthest to origin at the end of the experiment significantly increased when compared to 35%, reaching values even greater than control.

### 3.6. Apoptosis

**Figure 6:**
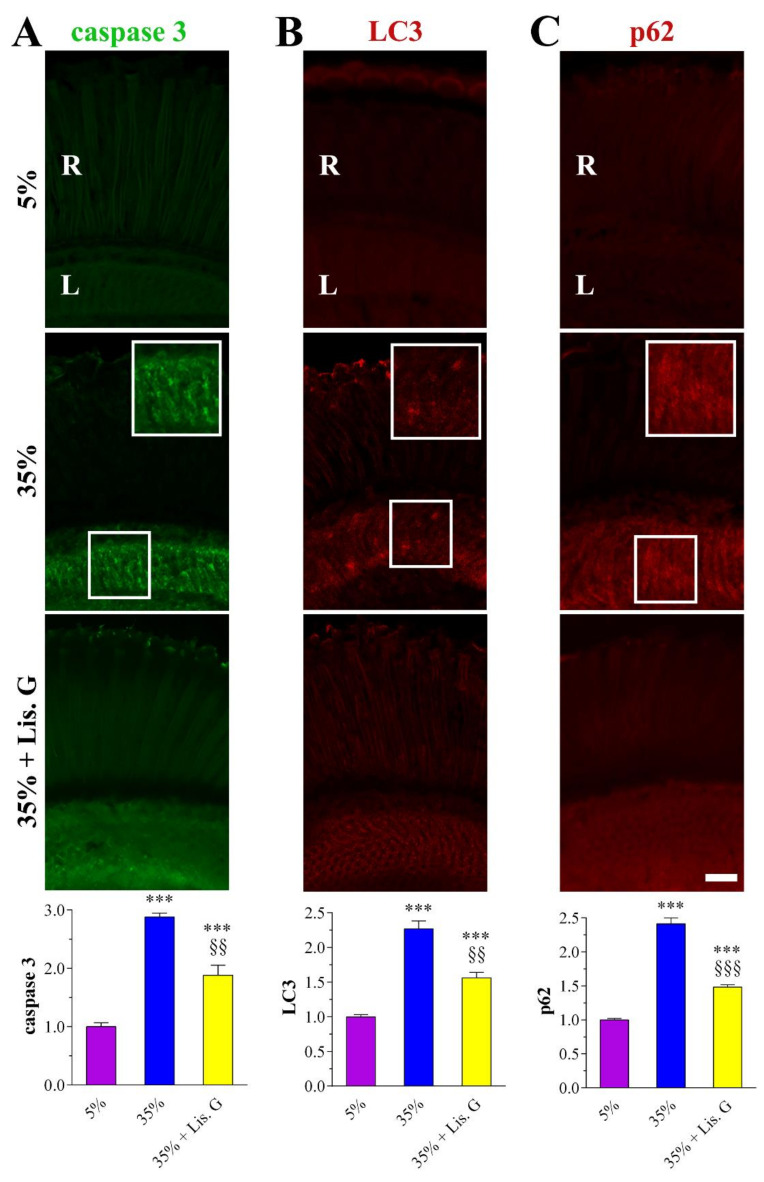
Apoptosis and autophagy in adult retina of *D. melanogaster* after 10 days feeding with 5% sucrose diet (control, normoglycemic flies) or supplemented with 35% sucrose (hyperglycemic flies), in the absence and in the presence of Lisosan G at 10 µg/mL. Confocal microscopy immunofluorescence imaging of (**A**) cleaved (active) caspase 3, (**B**) LC3, and (**C**) p62. Scale bars: 20 μm. Inserts (white boxes) represent enlarged image detail. R: retina, L: lamina. The graphs below depict the quantitative analysis of immunofluorescence staining. Results are expressed by setting the staining of 5% control as 1. *** *p* < 0.0001 vs. 5% control; §§ *p* < 0.001 and §§§ *p* < 0.0001 vs. 35% sucrose. Images and data are representative of at least *n* = 30 animals obtained from 5 independent experiments.

The hyperglycemic eye of adult *D. melanogaster* raised in high-sucrose diets displayed typical neurodegenerative features, namely apoptosis and dysfunctional autophagy flux, primarily affecting the internal network of the retina [22]. As shown in Figure 6A, the confocal microscopy immunofluorescence intensity of cleaved (active) caspase 3 as a marker of the apoptotic fly eye [20,22] remarkably increased in the lamina of flies fed for 10 days with 35% sucrose, while caspase 3 fluorescent aggregates reduced significantly in the presence of 10 µg/mL Lisosan G.

### 3.7. Autophagy

Similarly, the analysis of LC3 and p62 autophagy proteins in 35% sucrose-treated flies revealed a large amount of LC3/p62 clusters in the lamina when compared with 5% normoglycemic control [22], while their immunostaining was milder in the Lisosan G group (Figure 6B,C). Accordingly, quantitative analysis showed a significant decrease of LC3 and p62 expression in eyes sections of high-sucrose-fed Drosophila treated with Lisosan G, thus indicating a restoration of the autophagosome turnover close to basal levels.

**Figure 7:**
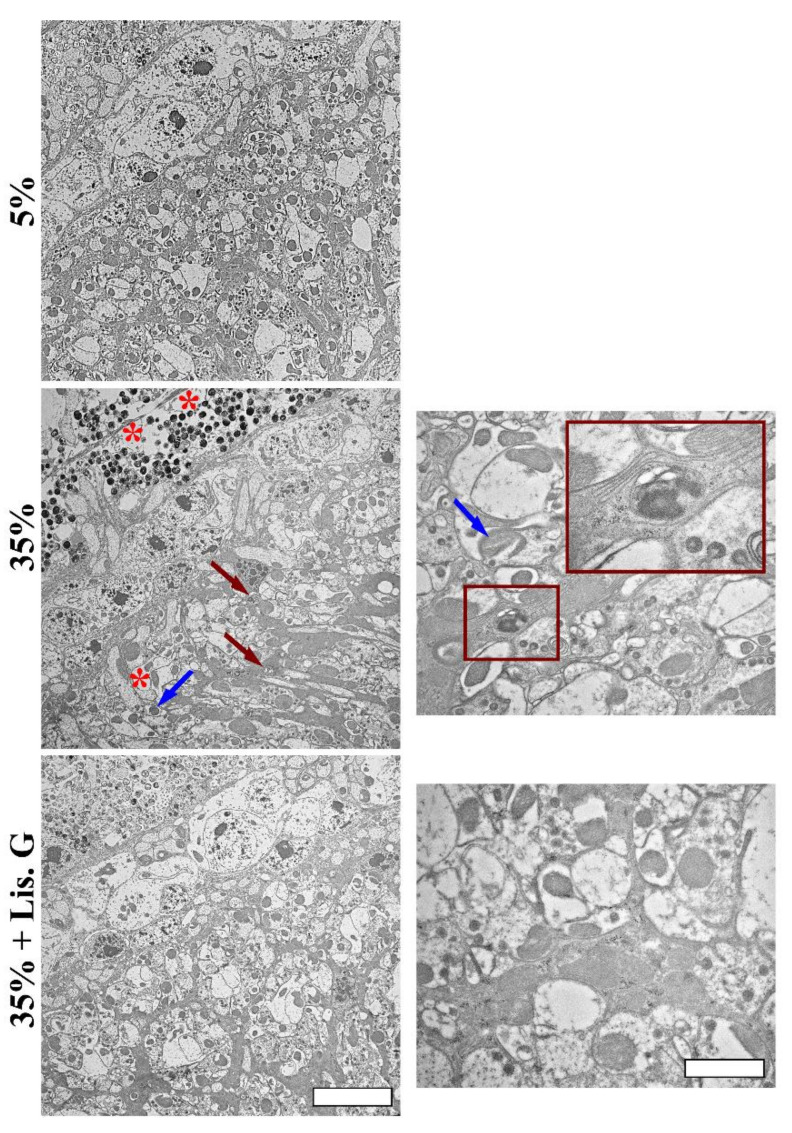
Eye ultrastructure by TEM analysis in adult retina of *D. melanogaster* after 10 days feeding with 5% sucrose diet (control, normoglycemic flies) or supplemented with 35% sucrose (hyperglycemic flies), in the absence and in the presence of Lisosan G at 10 µg/mL. Micrographs show the presence of accumulated autophagosomes (red arrows), vacuoles (red asterisks), and damaged mitochondria (blue arrows) in the lamina of 35% sucrose. The right panels represent higher magnification of different fields of 35% sucrose and 35% sucrose + Lisosan G samples. Insert (red box) represents enlarged image details of autophagosomes structure. Scale bars: 5 μm (left panels) and 2 μm (right panels). Images are representative of at least *n* = 10 animals obtained from 4 independent experiments.

In this respect, the accumulated autophagic vesicles with double or multiple-membrane containing electron-dense material [22], vacuoles, and damaged mitochondria were not evident in the lamina of flies treated with Lisosan G, as shown by TEM ultrastructural analysis (Figure 7).

### 3.8. Combined Effects of Lisosan G and Autophagy Activation

**Figure 8:**
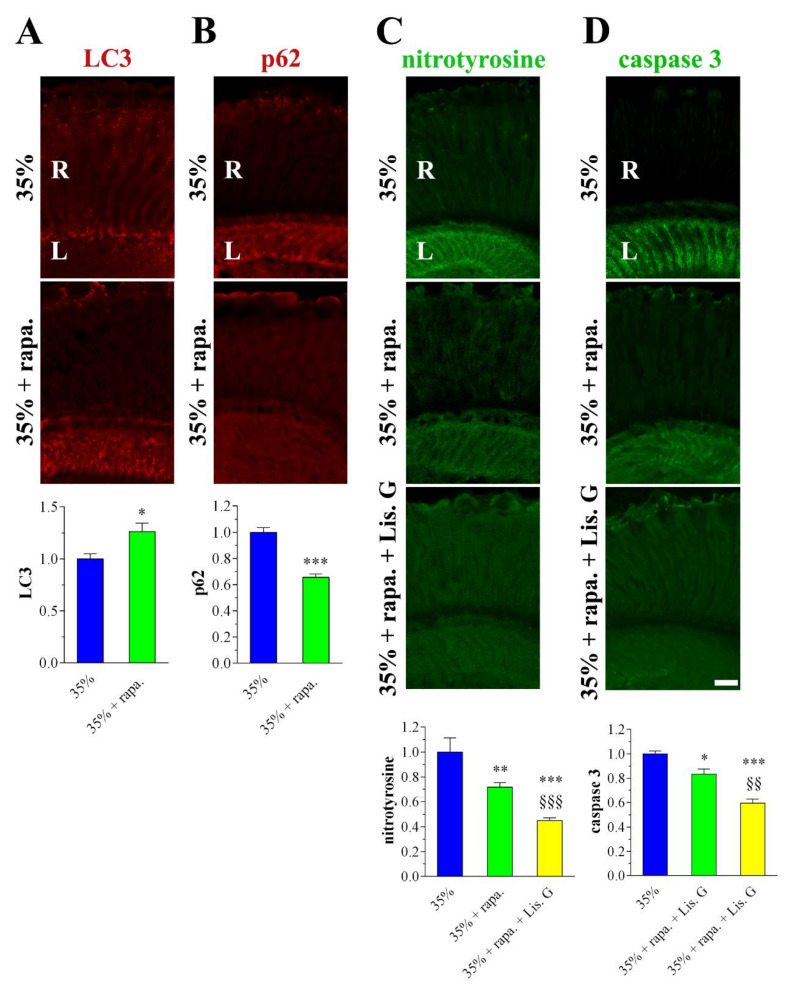
Autophagy, oxidative stress, and apoptosis in adult retina of *D. melanogaster* after 10 days feeding with 35% sucrose diet (hyperglycemic flies), in the absence and in the presence of rapamycin (10 nM) or Lisosan G (10 µg/mL). Confocal microscopy immunofluorescence imaging of (**A**) LC3, (**B**) p62, (**C**) nitrotyrosine, and (**D**) cleaved (active) caspase 3. Scale bars: 20 μm. R: retina, L: lamina. The graphs below depict the quantitative analysis of immunofluorescence stainings. Results are expressed by setting the staining of 35% sucrose as 1. * *p* < 0.05, ** *p* < 0.001, and *** *p* < 0.0001 vs. 35% sucrose; §§ *p* < 0.001 and §§§ *p* < 0.0001 vs. 35% sucrose + rapamycin. Images and data are representative of at least *n* = 20 animals obtained from 4 independent experiments.

In another set of experiments, 35% sucrose-treated flies were reared for 10 days on a diet supplemented with rapamycin, a widely used autophagy activator. Rapamycin was used at the optimum concentration of 10 nM, already shown to be effective in Drosophila eyes [20]. As expected, rapamycin increased further the expression/clustering of LC3 immunofluorescence in the lamina while p62 staining significantly decreased (Figure 8A,B). This indicated that rapamycin treatment can effectively boost the autophagy in retina neurons of hyperglycemic animals. As shown in Figure 8C,D, nitrotyrosine and active caspase 3 levels were reduced in 35% sucrose-treated flies fed with rapamycin, and, of interest, the administration of Lisosan G (10 µg/mL, 10 days) displayed additive inhibitory effects on both ROS generation and apoptosis.

**Figure 9:**
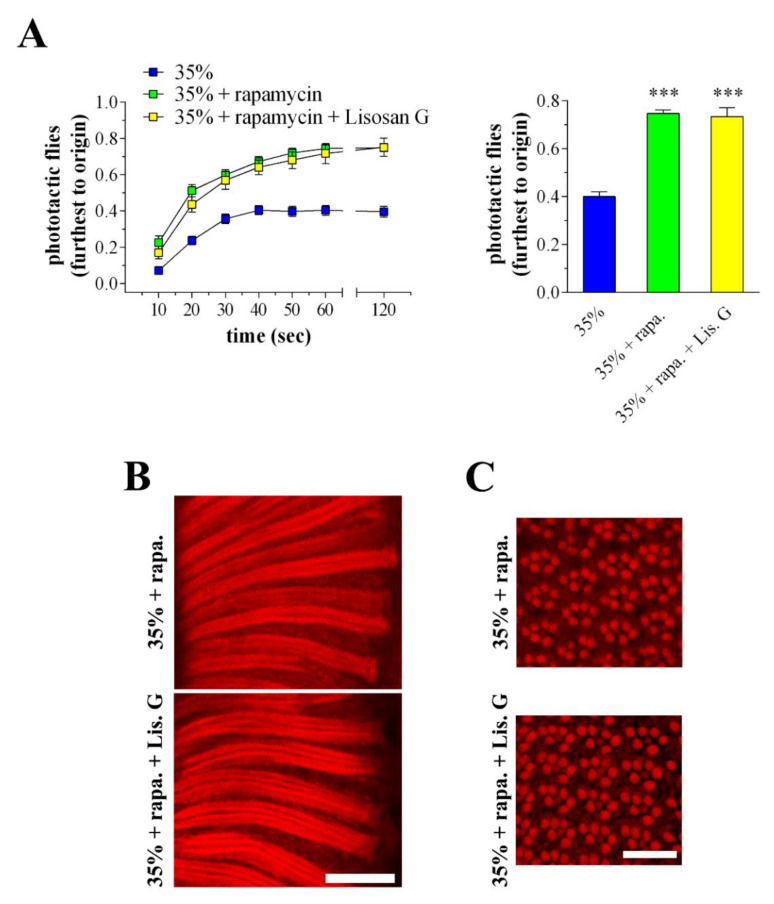
Phototaxis assay and eye structure of adult *D. melanogaster* after 10 days feeding with 35% sucrose diet (hyperglycemic flies), in the absence and in the presence of rapamycin (10 nM) or Lisosan G (10 µg/mL). (**A**) In the left panel, Drosophila were counted at 10, 20, 30, 40, 50, 60, and 120 s in the chamber furthest to origin (21–30 cm). In the right panel, Drosophila were analysed in the chamber furthest to origin at 60-120 s. Results are expressed as the percentage of total flies in the chambers at each time points. *** *p* < 0.0001 vs. 35% sucrose. Data are representative of at least *n* = 80 animals obtained from 4 independent experiments run in triplicate. (**B**) Conventional (longitudinal sections) and (**C**) confocal (cross sections) microscopy analysis of Drosophila eyes stained with fluorescent phalloidin (F-actin staining) to detect rhabdomere morphology and the pattern of ommatidia and rhabdomeres, respectively. Scale bars: 20 μm (longitudinal) and 10 μm (cross). Images are representative of at least *n* = 20 animals obtained from 4 independent experiments.

Moreover, autophagy activation improved the responsiveness to the light of hyperglycemic Drosophila (Figure 9A). In particular, flies raised under a 35% sucrose regimen in the presence of rapamycin reached the light with high efficiency during the phototaxis assay. Comparable results on Drosophila photosensitivity were found in the rapamycin + Lisosan G-treated group.

Accordingly, in the 35% sucrose + rapamycin-treated flies, the array of ommatidia and rhabdomeres in longitudinal and cross eye sections appeared similarly restored both in the absence and in the presence of Lisosan G (Figure 9B,C).

## 4. Discussion

Previous observations in adult flies fed for 10 days with 35% sucrose diets showed a clear decrease in light responsiveness as a consequence of vision defects [22]. Hyperglycemia did not alter the gross anatomical architecture of the external eye phenotype, although progressive damage of photosensitive units was reported. In addition, the retina internal network of Drosophila administered with 35% sucrose displayed typical neurodegenerative features, namely apoptosis, oxidative stress, and dysfunctional autophagy flux, thus offering a meaningful opportunity to study the visual alterations and the retina neurodegenerative hallmarks developed in patients at the early stages of DR, as well as to develop and test strategies counteracting hyperglycemic insult in the eye [22]. Noteworthy, the internal organs of flies are bathed in hemolymph without a vascular network to separate the blood cells from other tissues. This open circulatory system allows an investigation of neuroretinal alterations independently of the typical microvasculature and major inflammatory defects, which are related to the severity of DR complications [7].

### 4.1. Lisosan G and Eye Neurodegeneration

In streptozotocin-induced diabetic rats, the natural substance Lisosan G, commercialized as a nutritional supplement, showed anti-inflammatory effects when administered for oral gavage, with protective actions against neural/functional and vascular defects characteristic of DR [34]. Our in vivo study in *D. melanogaster* provides further evidence that Lisosan G has potential nutraceutical profiles against hyperglycemia-induced degeneration of retinal neurons. There is a general agreement that nutrients could offer a new line of defense against DR, with the efficacy of different formulations and administration as a key aspect to be considered [48]. In this respect, we demonstrated for the first time that food supplementation with Lisosan G positively affects the visual system, which is clearly damaged by hyperglycemia in high-sucrose-fed animals. In particular, Drosophila eye-structure injuries, i.e., defects of the standard pattern of ommatidia, irregular rhabdomeres, and clear alterations of phototransduction units, were, at least in part, rescued by Lisosan G administration. Comparably with other nutraceuticals with documented beneficial effects in rodent models of DR [49,50], Lisosan G exerted protective actions independent from glucose content and body weight, as reported in diabetic rats [34].

At the functional level, Lisosan G-supplemented food ameliorated the visual response of 35% sucrose-treated Drosophila and decreased the presence of cell death/apoptotic features in the retina internal network. These data point to the correlation between the anti-apoptotic effects of Lisosan G and functional recovery. Besides, we provide further mechanistic aspects since Lisosan G reactivated autophagy in the retina of hyperglycemic flies. Accordingly, typical hallmarks of neuroretina degeneration, for instance, vacuoles and damaged mitochondria, were not detectable at the ultrastructural level in eye neurons. Cell death-suppressing and differentiating effects of autophagy are required for Drosophila eye formation [51]. Also, defects in the autophagic pathway of Drosophila photoreceptors were involved in retinal degeneration [52]. The altered balance of apoptosis and autophagy plays a crucial (and well conserved) role in visual system damages across species [11,13,20,53]. There is evidence that enhanced autophagy could protect retinal cells under diabetic conditions by inhibiting apoptosis induced by DR stress [21]. Since neuronal defects and altered apoptosis/autophagy machinery has been observed in our system [22] as well as in the mouse retina of early DR and oxygen-induced retinopathy models [17,19], we suggest that proper autophagy induced by Lisosan G is a key aspect in the hyperglycemic fly eye to counteract neurodegenerative/functional features. Notably, in this scenario, sustained autophagosome turnover by rapamycin prevented retinal cell death and structural changes in 35% sucrose-treated Drosophila and ameliorated their light response. Similar results were achieved in dystrophic Drosophila mutants where the unbalanced autophagy turnover is responsible for retinal damage and functional alterations caused by defective full-length dystrophin [20].

### 4.2. Antioxidant Activity of Lisosan G

Among human tissues, the retina is one of the highest consumers of oxygen and is prone to oxidant burden. Aberrant ROS are responsible for retinal injury and a common feature of retinal neurons in DR [6,7,8,9,11]. Decrease of oxidative stress and activation of antioxidant/detoxifying enzymes were observed in Lisosan G-treated cells [29,30,31,32], as well as its radical scavenger activity in acellular systems [28,32]. In mouse retina explants challenged with oxidative stress, Lisosan G affected the mRNA expression of some antioxidant enzymes and the nuclear translocation of Nrf2, the redox-sensitive transcription factor, in the retina of streptozotocin-treated rats, although these data claiming the antioxidant role of Lisosan G in neurons were not conclusive [34]. Our results clearly showed the antioxidant effects of Lisosan G in 35% sucrose-treated Drosophila. In particular, food supplementation with Lisosan G reduced brain ROS and retina peroxynitrite levels. Of note, peroxynitrite is a powerful oxidant found to be elevated in the retina early in diabetes and to couple with retinal neurodegeneration in experimental and human diabetes/hyperglycemic status [22,54,55,56].

In our system, we also attempted to profile the mechanisms of oxidative stress-related perturbations in the Drosophila brain tissue at the level of metabolite composition. Among the considerably accumulated metabolites in hyperglycemic flies, we detected quinolate, which represents one of the most neurotoxic metabolites causing the formation of lipid peroxidation products in nervous tissue [57]. Toxic cascades triggered or stimulated by quinolate involve the formation of both ROS and reactive nitrogen species , thus leading cells to oxidative damage as part of their degenerative processes [58]. The considerable lowering of quinolate levels in 35% sucrose-treated Drosophila administered with Lisosan G may thus indicate the prevention of quinolinic acid-induced neuronal damage. DHO metabolite is involved in the de novo pyrimidine synthesis pathway, where DHO is oxidized to orotate in a reversible reaction catalyzed by dihydroorotate dehydrogenase (DHODH), the single redox step in pyrimidine synthesis. In fact, DHODH is the only enzyme in the pyrimidine biosynthesis that is located in the mitochondria rather than in the cytosol, where it affects ROS production [59]. The strong down-regulation of DHO in 35% sucrose-fed fly samples suggests a high rate of conversion of this metabolite towards orotate production; this DHO oxidation may drive superoxide/H_2_O_2_ generation enough to have detrimental cellular effects as previously reported [60]. We observed a restoration of DHO levels in hyperglycemic flies fed with Lisosan G and, consequently, a plausible normalization of the endogenous ROS levels. In addition, hyperglycemic conditions decreased the GSH amount in the Drosophila brain tissue with a consequent reduction of the GSH:GSSG ratio. This led to a loss of the antioxidant capacity of the glutathione system, thus impairing the non-enzymatic antioxidant defenses in the brain. On the other hand, these events were inhibited when the 35% sucrose fly diet was supplemented with Lisosan G, further demonstrating its antioxidant role. Accordingly, Lisosan G increased GSH in hepatocytes of carbon tetrachloride-intoxicated rats [28]. GGSH is the primary antioxidant responsible for maintaining the reducing intracellular microenvironment that is essential for normal cellular function and viability. Neurons are particularly vulnerable to excess ROS, hence requiring continuous supply and regeneration of GSH [61]. Hyperglycemic Drosophila also displayed low levels of γ-GC. This down-regulation affects the ratio of GSH:GSSG because γ-GC is the most immediate precursor of GSH, directly acting as a substrate of GSH synthetase. Of interest, γ-GC levels strongly up-regulated in 35% sucrose flies after food supplementation with Lisosan G, thus sustaining GSH content of cells with consequent attenuation of ROS-induced damage. In this respect, endogenously synthesized γ-GC exerted neuroprotection in primary neurons and in an in vivo mouse model of neurodegeneration [62]. Moreover, γ-GC was shown to ameliorate oxidative injury in neurons and astrocytes and to increase brain GSH levels [63]. Not only GSH but also the dipeptide Cys-Gly is considered one of the most important low molecular-mass sulfhydryls occurring in the intracellular milieu. These molecules are metabolically interrelated and are crucial in determining the redox environment and free radical interactions. Cys-Gly is an intermediate of GSH turnover: it is hydrolyzed, upon entry into neurons, by an ectopeptidase, thus providing cysteine and glycine as precursors for GSH synthesis [64]. Our data revealed a drop in Cys-Gly abundance in 35% sucrose-treated Drosophila fed with Lisosan G, corroborating the mechanistic insights of Lisosan G-induced GSH levels. Moreover, we suggest a relationship between 7,8-DHF, uric acid, and allantoin. In detail, 7,8-DHF may be metabolized in hyperglycemic flies with the final production of uric acid [65], which resulted significantly accumulated while allantoin levels decreased. Uric acid acts as both an antioxidant and a pro-oxidant molecule [66]. Unlike humans, in most species, such as *D. melanogaster*, uric acid is metabolized to allantoin by the enzyme urate oxidase [67]. In urate oxidase-knockdown cells of flies, uric acid has been shown to generate a pro-oxidative environment with a significant increase in ROS levels [67]. Interestingly, uric acid levels decreased in 35% sucrose-treated Drosophila fed with Lisosan G, whereas allantoin accumulated. These results support the hypothesis that Lisosan G reduces oxidative stress also by inducing the conversion of uric acid to allantoin, likely through the activation of urate oxidase.

Emerging evidence reveals that oxidative stress-induced disruption of autophagy in retinal cells is closely related to the pathogenesis of DR [21]. In this context, autophagy can prevent cells from oxidative stress by diminishing harmful intracellular materials. Accordingly, our results showed that the increase of autophagosome turnover by rapamycin prevents peroxynitrite formation and damage in 35% sucrose-treated Drosophila eyes. Of interest, the food supplementation with Lisosan G (which restores baseline autophagy) and rapamycin (which maximizes autophagic turnover) displayed additive inhibitory effects on retinal ROS production and apoptosis, thus suggesting a possible inhibition of oxidative stress by Lisosan G and, consequently, retinal cell death of hyperglycemic flies, also independently from autophagy. However, autophagy-ROS cross-talk seems to play a key neuroprotective role since Lisosan G and rapamycin, both alone and in combination, reached comparable positive effects on structural and functional features of the hyperglycemic fly eye, indicative of multiple and redundant actions of Lisosan G.

## 5. Conclusions

Exogenous antioxidants of natural origin may be used to preserve redox homeostasis in the diabetic retina [25,26,27]. They may act directly as scavengers of free radicals, indirectly by interrupting free radical chain reactions, or both. They may also decrease oxidative stress by inducing the expression of endogenous antioxidant enzymes. Oral supplementation with natural antioxidants carries the great advantage of being a non-invasive treatment with presumably no harmful effects. The role of oral antioxidants has provided promising results in retinal diseases, such as age-related macular degeneration [68], although they have not been included yet as a routine treatment for DR patients [27].

**Figure 10:**
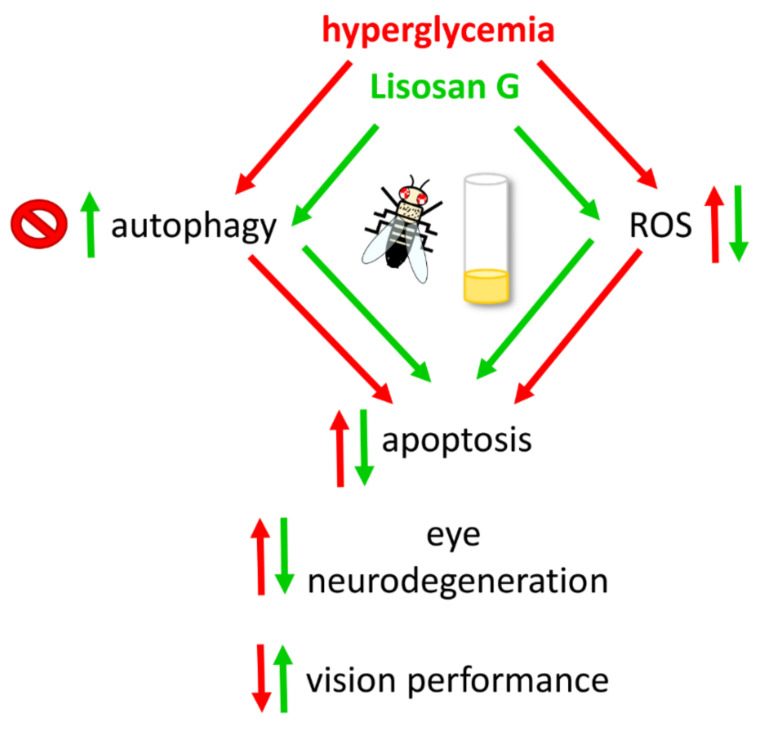
Schematic picture depicting the neuroprotective mechanisms of Lisosan G on the hyperglycemic eye of adult *D. melanogaster*.

*D. melanogaster* is considered a very potent in vivo tool to study human neurodegenerative diseases, including eye conditions, to complement more traditional vertebrate systems [23,69,70,71]. In this respect, we recently reported retinal neuron damage and synapse alterations in flies carrying full-length dystrophin defects, which paralleled well with the results obtained in *mdx* mice, the most commonly employed models in Duchenne muscular dystrophy research [20]. Drosophila has the significant strength of allowing specific expression experiments in the context of a powerful and well-established genetic framework, also providing a great advantage concerning animal husbandry and the short generation time and lifespan. As summarized in Figure 10, this study demonstrated that a continuous supplementation of Lisosan G with diet exerts a robust and multifaceted antioxidant effect on fly retinal neurons, thus providing efficacious neuroprotection (pro-autophagic and anti-apoptotic actions) against high-glucose-induced eye damage. Being aware that pre-clinical pharmacological experiments are required in multiple models to ascertain both histopathology and visual function, we suggest that Lisosan G nutraceutical-based approach may be an economical and sustainable treatment that deserves to be investigated in DR therapeutic strategies.

## Figures and Tables

**Table 1:** Results of one-way ANOVA with Fisher’s LSD test.

Metabolites	f-Value	*p*-Value	-log10(p)	FDR	Fisher’s LSD *
2-aminooctanoic acid	20.148	0.002177	2.6622	0.0208	3-1; 3-2
3-S-methylthiopropionate	14.43	0.005099	2.2925	0.0367	2-1; 3-1
5-hydroxykynurenamine	60.315	0.000106	3.9732	0.0038	3-1; 3-2
7,8-dihydrofolate	14.479	0.005056	2.2962	0.0367	3-1; 3-2
allantoin	20.855	0.001989	2.7014	0.0204	1-2; 3-2
anthranilate	357.56	5.76 × 10^−7^	6.2396	4.15 × 10^−5^	2-1; 3-1; 3-2
ascorbic acid	13.619	0.005882	2.2305	0.0403	2-1; 3-1
Cys-Gly	21.665	0.001799	2.7449	0.0204	2-1; 2-3
D-erythrose-4-phosphate	18.753	0.002623	2.5812	0.0236	2-1; 2-3
dihydroorotate	21.232	0.001898	2.7218	0.0204	1-2; 3-2
dTMP	34.156	0.000526	3.2787	0.0084	2-1; 2-3
methylcysteine	38.627	0.000374	3.4268	0.0074	1-2; 3-1;3-2
methylmalonic acid	37.26	0.000414	3.3832	0.0074	1-2; 3-2
myo-inositol	16.086	0.003883	2.4108	0.0310	3-1; 3-2
N-acetyl-L-ornithine	22.46	0.001636	2.7862	0.0204	1-2; 1-3
p-aminobenzoate	357.56	5.76 × 10^−7^	6.2396	4.15 × 10^−5^	2-1; 3-1; 3-2
quinolate	41.163	0.000313	3.5038	0.0074	2-1; 2-3
succinate	37.26	0.000414	3.3832	0.0074	1-2; 3-2
uric acid	16.168	0.003834	2.4163	0.0310	2-1; 2-3
xanthine	32.804	0.000588	3.2304	0.0084	2-1; 3-1
γ-L-glutamyl-L-cysteine	224.41	2.30 × 10^−6^	5.639	0.0001	1-2; 3-1;3-2

* Adult *D. melanogaster* after 10 days feeding with: 1 = 5% sucrose diet (control, normoglycemic flies); 2 = 35% sucrose diet (hyperglycemic flies); 3 = 35% sucrose diet + Lisosan G (10 µg/mL). Data are representative of 3 replicate samples from at least *n* = 100 animals.

## Data Availability

Data is contained within the article and Supplementary Materials.

## Supplementary Materials

The following are available online at https://www.mdpi.com/article/10.3390/antiox10081197/s1, Figure S1: body weight (**A**) and whole-body glucose (**B**), Figure S2: changes of GSH and GSSG levels in adult *D. melanogaster* after 10 days feeding with different diet regimens.
