# Supplementary material for: Nutraceutical Strategy to Counteract Eye Neurodegeneration and Oxidative Stress in Drosophila melanogaster Fed with High-Sugar Diet
Source: Antioxidants (Basel). 2021 Jul 27;10(8):1197. doi: 10.3390/antiox10081197 (PMC8388935; doi:10.3390/antiox10081197)
Supplement: Supplementary file 1 [file antioxidants-10-01197-s001.zip › antioxidants-1303537-supplementary.pdf]

Supplementary Figure S1

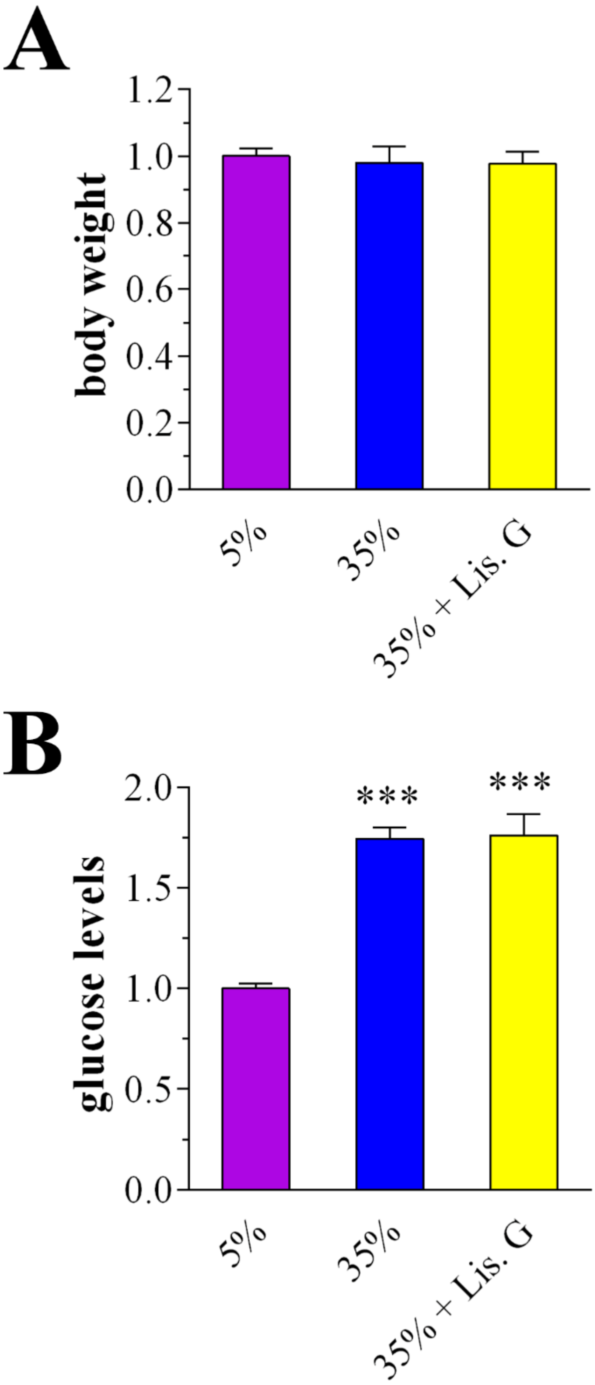

Figure S1. Body weight **(A)** and whole-body glucose **(B)** in adult *D. melanogaster* after 10 days feeding with 5% sucrose diet (control, normoglycemic flies) or supplemented with 35% sucrose (hyperglycemic flies), in the absence and in the presence of Lisosan G at 10 µg/ml. Glucose was measured by means of Liquid chromatography-Mass spectrometry and related to the fly weight. Results are expressed by setting the values of 5% sucrose as 1. *** $p < 0.0001$ vs 5% control. Data are representative of at least n = 60 animals obtained from 3 independent experiments.

Supplementary Figure S2

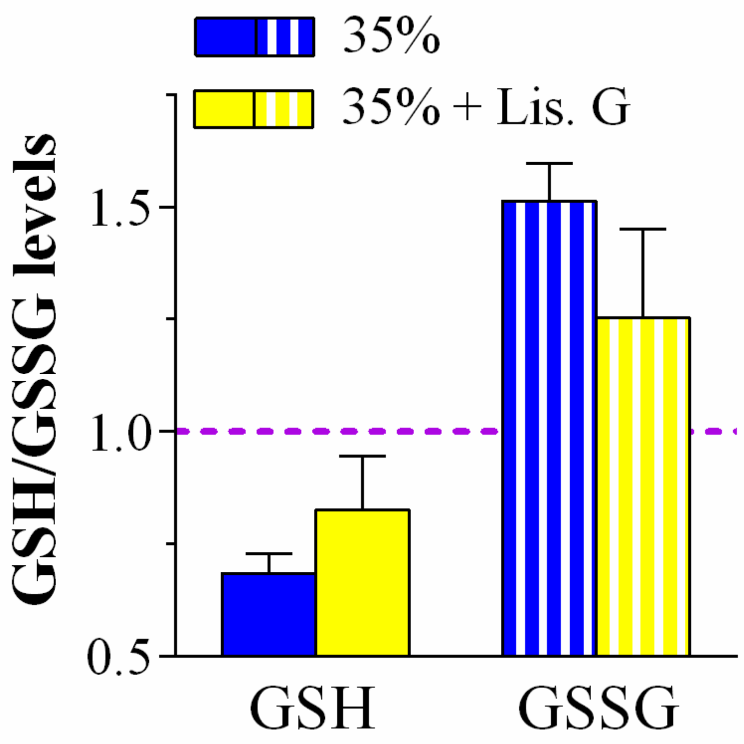

Figure S2. Changes of GSH and GSSG levels in adult heads of *D. melanogaster* after 10 days feeding with 35% sucrose diet (hyperglycemic flies), in the absence and in the presence of Lisosan G at 10 μg/ml. Results are expressed by setting the values of 5% sucrose (control, normoglycemic flies) as 1 (purpled dotted line). Data are representative of 3 replicate samples from at least n = 100 animals.
